# Men and Women as Differential Social Barometers: Gender Effects of Perceived Friend Support on the Neuroticism-Loneliness-Well-Being Relationship in a Younger Adult Population

**DOI:** 10.3390/ijerph19137986

**Published:** 2022-06-29

**Authors:** Julie M. Turner-Cobb, Emily Arden-Close, Emma Portch, Liam Wignall

**Affiliations:** Department of Psychology, Bournemouth University, Poole, Dorset BH12 5BB, UK; eardenclose@bournemouth.ac.uk (E.A.-C.); eportch@bournemouth.ac.uk (E.P.); lwignall@bournemouth.ac.uk (L.W.)

**Keywords:** loneliness, social relationships, personality, neuroticism, health, well-being, gender, pandemic, COVID-19

## Abstract

Loneliness and social isolation are well known to have detrimental effects on mental and physical health, and the perception of social support is frequently viewed as a protective factor. Yet, the beneficial effect varies when perceived support is considered with respect to gender and personality. We examined the mechanism of loneliness as a mediator of personality on health and moderation of this relationship by perceived social support and gender. Five hundred and thirty young adults (325 women) aged 18–32 years (Mage = 25.42, SD = 4.13) provided self-report assessments of personality, loneliness, perceived social support, general health and psychological impact of the COVID-19 pandemic on well-being. Using a series of regression-based mediation and moderated mediation models, we found higher scores on extraversion to be associated with lower loneliness and better general health and well-being; higher neuroticism with greater loneliness and poorer general health. Being male and perceiving greater friend support moderated the neuroticism–loneliness–well-being relationship. Men higher on neuroticism were less able to benefit from lower loneliness when the perception of support from friends was greater, yet were less sensitive to the negative impact on the well-being of perceiving low levels of friend support. Effects suggest important gender differences with the potential to inform health interventions.

## 1. Introduction

Social support, described as support an individual can access through social connections to others, groups and the larger community [1], has consistently been associated with positive health-related outcomes and its absence with negative ones, whether in the naturalistic environment or in the laboratory [2,3] and can lead to increases in morbidity and mortality [4]. As a protective factor against loneliness [5], social support has been found to mediate the relationship between loneliness and life satisfaction [6]. According to social safety theory [7], the development and maintenance of social bonds is a fundamental principle of human behaviour, with loneliness and social isolation conceptualised as threats to social safety, leading to changes in disease risk, cognitions and behaviours.

When the World Health Organization declared a pandemic due to COVID-19 [8], governments around the world responded with the implementation of various social restrictions to curb the spread of the coronavirus. These included “stay at home” or “lockdown” restrictions and were considered effective due to ease of viral transmission in enclosed spaces or crowded areas [9,10]. From a public health perspective, physical separation of people is an effective strategy to prevent the spread of infectious diseases [11]. Yet, it is also known that suppression of face-to-face interactions can erode social bonds, leading to increased loneliness with significant negative impacts on psychological health [12,13]. Data from a large UK epidemiological study suggests that people with the highest levels of loneliness at the start of lockdown had greater increases in loneliness across subsequent weeks [14]. Under social lockdown conditions designed to facilitate physical safety, the notion of social safety provides a conundrum since the usual positive social connections naturally sought to provide protection become potential threats for viral transmission and access to available social support is impacted. 

A substantial body of literature supports loneliness being associated with impaired mental and physical well-being [15,16]. Recent work examining mental health during the COVID-19 pandemic found higher levels of stress, depression and anxiety symptoms worldwide [17], including in the UK [18] and the United States [19,20]. During the pandemic, there has been an increased risk of loneliness, particularly in women, young adults and those with lower educational attainment [21] and increased suicidal ideation [22]. Within the social climate of the pandemic, social support has been identified as a buffer against the negative impact of low resilience on mental health [23] and as a mediator of stressful experiences on acute stress disorder symptoms [24].

Conversely, evidence suggests that negative social interactions, which involve an element of criticism or impose excessive demands, negatively impact psychological well-being [25,26,27]. Perception of negative feelings such as loneliness also mediates the effect of social support on subjective well-being [28]. Loneliness can reflect a discrepancy between desired and actual relationships, both of which may be influenced by personality [29]. Of the Big Five personality traits [30], loneliness appears to be most strongly associated with neuroticism (characterised by heightened sensitivity to negative social stimuli) and Extraversion (characterised by heightened sensitivity to positive social stimuli) [31]. Evidence exists that shows neuroticism and loneliness to be highly correlated genetically and for a causal relationship to exist in the direction of neuroticism increasing loneliness [31]. In a longitudinal analysis of reciprocal relationships between neuroticism, loneliness and subjectively reported general health, neuroticism not only predicted poorer health and greater loneliness but subjective health and loneliness both predicted future neuroticism scores [32]. It is, therefore, important to include the role of personality in examining the effect of loneliness on physical and mental well-being and in relation to other psychosocial and demographic factors that might interplay in this relationship, particularly when a social threat may be exposed under conditions of mandated lockdown such as those experienced in the COVID-19 pandemic. 

Perceived social support has been demonstrated to influence coping mechanisms [33], reducing distress experienced during natural disasters [34] or trauma [35]. When assessing the potential buffering role of social support, perceived levels were a stronger predictor than objective received levels of social support [36]; perceived support was more strongly correlated with loneliness than received and structural support [37]. Individuals with high levels of neuroticism or extraversion who perceive a lack of available or accessible social support during lockdown might experience poorer health than those who perceive themselves as having strong social support. 

Gender differences also exist in relation to sources and perceptions of social support. Women are more likely to nominate friends as social networks, whilst men tend to name their partners as their main source of support [38,39]. Yet, in clinical populations, such as patients with depressive disorder, the opposite pattern has been found, with men perceiving greater support from friends and women from their significant other [40]. Furthermore, others have found that females give and receive more social support than males on social networking sites [41]. There are also gender differences in the moderating effects of perceived social support, with evidence to suggest that the health benefits of social support are greater for men than women. For example, whilst marriage has been found to be protective against mortality, it appears to have greater benefits for men [42], and the presence of a supportive male friend has been found to reduce cardiovascular reactivity in response to acute stress testing [43]. In women, family support, rather than friend or spousal support, has been significantly linked to reduced mortality risk, whilst friend or spouse strain has been linked to increased mortality risk [44]. 

The current study aimed to examine psychosocial mechanisms of loneliness and perceptions of social support through which personality might influence self-reported health and well-being under the microscope of conditions brought about by the pandemic lockdown. Specifically, our objectives were to systematically assess the indirect effect of loneliness on the relationship between personality characteristics on health during the initial coronavirus lockdown in the UK and to evaluate perceived support and the role of gender in these relationships. We hypothesised that feelings of loneliness would mediate the relationship between personality and health, that social support would act as a buffer of direct and indirect effects to influence personality on self-reported health, and that differentiation by gender would be observed in which social support benefitted men to a greater degree than women.

## 2. Materials and Methods

### 2.1. Participants and Recruitment

A sample of five hundred and thirty younger adults aged 18–32 years (Mage = 25.42, SD = 4.13, 61.3% (*n* = 325) = female) was drawn from a broader project (N = 565) that examined impacts of social isolation during the pandemic on sexual well-being and intimacy [45,46] in participants living in the UK. Inclusion criteria for the broader study required participants to be aged 18–32 years and living in the UK at the point of data collection. The current sample included those for whom complete data were available on all variables of interest for the present analysis and excluded 35 participants (six due to missing data on health DVs, 24 on IVs and one on gender, plus four participants who identified as non-binary). Anonymised study data are stored in Bournemouth University’s Online Research Data Repository (BORDaR). Almost half of the sample were educated to a degree level, over 80% were of White ethnicity, the majority (79.6%) lived with a partner or family member(s) and 30.2% were self-isolating at the time of the study (see Table 1). Age was not significantly correlated with either of the outcome variables. Significant differences were seen for education (*p* < 0.001) and self-isolation (*p* = 0.004) on self-reported general health (those in higher education groups and self-isolating reported better health) and for gender (*p* < 0.001) on impact on well-being (greater impact on well-being for women).

### 2.2. Measures

In addition to sociodemographic factors, self-report questionnaires were used to measure psychosocial factors and health as the key variables of interest.

Personality—Neuroticism and Extraversion. Twenty items, ten assessing extraversion (5 + vely keyed/5 − vely keyed) and ten assessing neuroticism (5 + vely keyed/5 − vely keyed), were sourced from the International Personality Item Pool (IPIP) [47] adapted from the NEO measures (IPIP, 2019) [48]. Items were scored from 1 (very inaccurate) to 5 (very accurate). There was good internal consistency in the current sample (Cronbach’s α = 0.91 extraversion/0.88 neuroticism).

Loneliness and Isolation. Assessed using the UCLA Loneliness Scale, Version 3 [49], composing of 20 items (e.g., How often do you feel alone? How often do you feel isolated from others?) rated on a 4-point scale (1 = never to 4 = always). This standardised scale has high internal consistency (α coefficients reported as 0.89 to 0.94) with robust validity [49]. Higher scores indicate greater feelings of loneliness. Cronbach’s α coefficient for the current sample was 0.93.

Perceived Social Support. The Multidimensional Scale of Perceived Social Support, (MSPSS) [50] consisting of 12 items (e.g., There is a special person who is around when I am in need, I can count on my friends when things go wrong, I can talk about my problems with my family), was used to generate a perceived total mean support score and three four-item subscales to assess perceived support from a significant other, family and friends. The MSPSS has good internal consistency (α coefficients 0.72 total score, and respective subscales 0.72, 0.85 and 0.75) and construct validity [50] across a range of populations, including young adults [51,52]. Internal consistency in the current sample was high for the total and three subscales (Cronbach’s α = 0.92 total, 0.96 significant other, 0.93 family and 0.94 friends).

Self-Rated Health and Well-Being. Two brief single-item questions were used as measures of physical health and mental well-being. The physical health item utilised the general health question derived from the Short Form Health Survey (SF-36) [53], previously used as a single item to measure self-rated health accurately [54]. There is substantial evidence for single-item health questions of this type showing strong predictive power with mortality even when controlling for other health conditions [55] and of association with physiological immune markers of inflammation [56]. This item asked participants to rate “In general, would you say your health is…” on a 5-point response scale (5 = excellent to 1 = poor). A higher score indicates better general overall health. Subjective mental well-being was assessed as having an impact on well-being in relation to the experience of the COVID-19 situation, with participants asked, “To what extent is the current pandemic impacting on your sense of well-being?” with responses on a scale from 0 (not impacting at all) to 100 (impacting a lot). A higher score indicated a greater impact on well-being (poorer mental health).

### 2.3. Procedure

The study received prior approval from the Science, Technology and Health Research Ethics Panel at Bournemouth University. Participants were recruited through Prolific, an online participant recruitment website used for academic surveys and market research. They provided written informed consent to participate and completed all study questions via the survey hosting website, Qualtrics. Data was collected between 14–18 May 2020, seven weeks into the first strict UK social lockdown protocol. The questionnaire took between 20–45 min to complete, with a reimbursement of £3.75 on data submission.

### 2.4. Statistical Analysis

**Figure 1:**
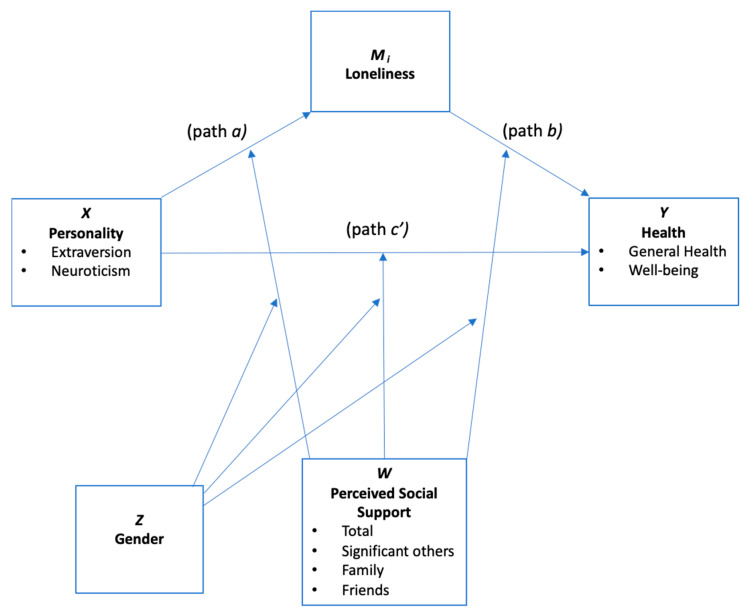
Conceptual moderated mediation model showing effects of personality on health via loneliness. Indirect effect of loneliness (mediator) by perceived social support (moderator) and gender (moderator) conceptualised to carry the effect of personality on health.

Preliminary analyses used *t*-tests for comparison of gender differences across psychosocial variables, and intercorrelations were examined disaggregated by gender. A series of regression-based analyses were then conducted to assess mediation and conditional moderated mediation effects using PROCESS [57] macro version 3.5.2 for SPSS (version 26.0). Mediation models (PROCESS model 4) examined the effect of loneliness on health with personality (extraversion, neuroticism) conceptualised as the independent variable (X), health (self-reported general health and impact on well-being) as the outcome (Y) and loneliness as the mediating variable (M). Separate models were conducted for the two health outcomes and two personality dimensions. Moderated mediation (PROCESS model 73) was then conducted to assess the indirect effects of personality on health via loneliness, conditional on the moderators of perceived support (W) and gender (Z) (Figure 1). Overall moderated mediation effects and three-way interactions (moderating effects of perceived social support and gender) on specific paths are reported. Independent models were run for each of the four support variables (total, significant others, family and friends) applied in combination with the health outcome and personality dimensions. All models adjusted for covariates of age, education level and isolating status.

Analyses were set at 5000 bootstrap samples, with mean centring of continuous variables that defined products and conditioning values for moderation as −1SD, mean and +1SD. Indirect and conditional effects of X on Y, with coefficient statistics of b (unstandardised beta), standard error of b (SE) and 95% confidence intervals used to interpret significance reported for each mediation, moderation and moderated mediation effect. Evidence of significance in simple mediation was assessed through the evaluation of indirect effects of X on Y and moderated mediation with social support and gender through the evaluation of pairwise contrasts between conditional indirect effects with interpretation of indirect effects of X on Y.

## 3. Results

### 3.1. Descriptive Analyses of Psychosocial Variables

Compared to men, women scored significantly higher on neuroticism and reported a significantly greater impact of the COVID-19 situation on their well-being. Men reported significantly lower perceived significant other and friend support compared to women (see Table 2). Intercorrelations disaggregated by gender revealed that for both genders, general health was significantly correlated with all psychosocial variables except perceived support from significant others for men (see Table 3). Impact on well-being was significantly correlated with neuroticism and loneliness for both genders; for women, a significant correlation was also observed between the impact on well-being and perceived support from family. The direction of effects was the same for both genders; extraversion and social support were positively associated with general health and negatively associated with impact on well-being, whilst neuroticism and loneliness were negatively associated with general health and positively linked to greater impact on well-being. For women, higher perceived support from all sources was associated with better general health and higher perceived family support was associated with less of an impact of the pandemic on well-being. However, for men, all perceived support variables, with the exception of support from significant others, were significantly correlated with general health, but none of the perceived support indices was associated with the impact of the pandemic on well-being.

### 3.2. Main Analyses

#### 3.2.1. Mediation: Loneliness as a Mediator of Personality on Health

A significant indirect effect of extraversion on self-reported general health through loneliness was observed (see Supporting Information Figure S1 panel A); a higher level of extraversion was associated with a lower level of loneliness (path a), and this lower level of loneliness was linked to better reported general health (path b). The effect of neuroticism on general health was mediated by loneliness (see Supporting Information Figure S1 panel B); a higher score on neuroticism was associated with a greater level of loneliness (path a), and this higher level of loneliness was linked to poorer reported general health (path b). A direct effect (c′ path) was also seen for neuroticism but not for extraversion on general health (see Supporting Information Figure S1 panels B/A respectively). For the impact of the pandemic on self-reported well-being, extraversion was mediated by loneliness (see Supporting Information Figure S1 panel C); the negative relationship observed in path *a* between greater extraversion and lower levels of loneliness were associated with less of an impact on well-being. A direct effect (c’ path) of extraversion on well-being impact was also noted (see Supporting Information Figure S1 panel C). There was no significant indirect effect of loneliness as a mediator of neuroticism on well-being impact, but a significant direct effect (c′) of higher neuroticism on greater well-being impact was observed (see Supporting Information Figure S1 panel D). Indirect effects were consistent in adjusted and unadjusted models.

#### 3.2.2. Moderated Mediation Analyses: Perceived Social Support and Gender as Moderators

With perceived social support (W) and gender (Z) as moderators in the mediation models with general health as the outcome, bootstrap confidence intervals of pairwise contrasts between conditional effects revealed no evidence in support of moderated mediation. Similarly, moderated mediation was not supported for models including extraversion (X) on well-being impact (Y). For models in which neuroticism (X) was the antecedent and impact on well-being (Y) was the outcome variable, support for a mediation effect (via loneliness) moderated by both perceived support and gender was found for perceived friend support but not for perceived total, significant other or family support (Supporting Information Table S1 shows pairwise contrasts). Mean and high but not low levels of perceived friend support moderated the neuroticism–loneliness–well-being relationship in men and not women (see Supporting Information Table S2 for conditional indirect effects). For men, a higher score on neuroticism was associated with a greater impact of the pandemic on well-being via loneliness when support from friends was perceived as higher. The full statistical model is given in Supporting Information Figure S2. Effects were consistent in unadjusted models with the exception of perceived support total, which reached significance only in the unadjusted model and showed the same pattern as found for perceived friend support.

Three-way interactions were also observed for this neuroticism–loneliness–well-being model, in which the moderators (perceived friend support and gender) influenced the relationship between variables on specific paths (Table 4).

**Figure 2:**
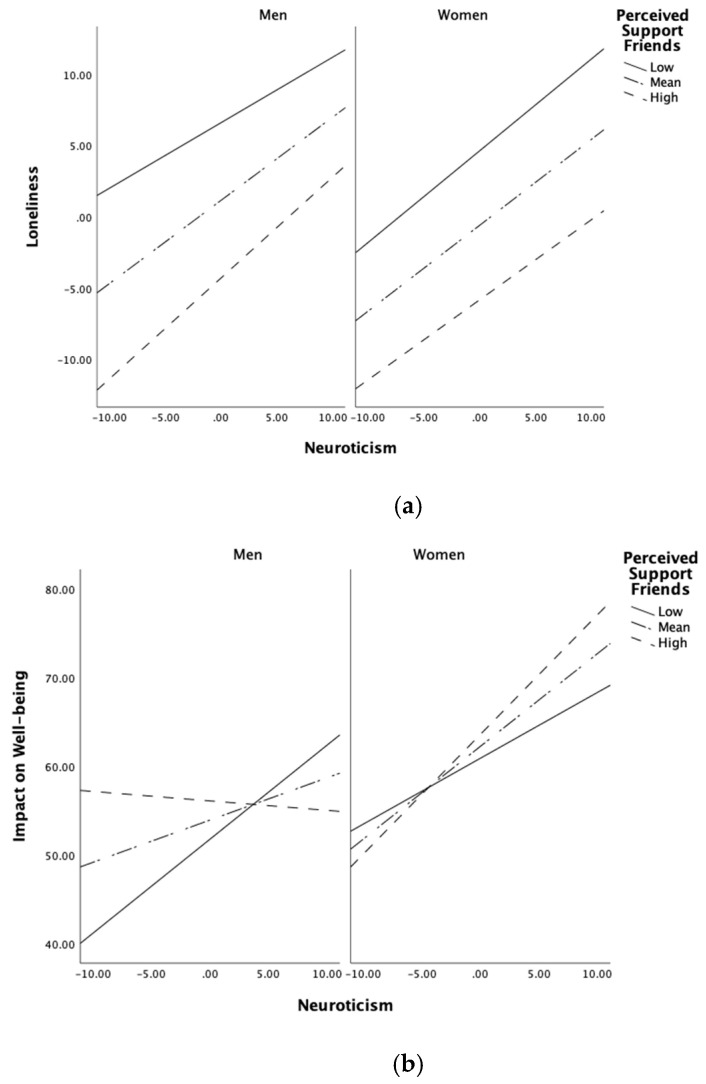
(**a**) Regression of loneliness on neuroticism at three levels of perceived support from friends by gender; (**b**) regression of well-being on neuroticism at three levels of perceived support from friends by gender. Values are plotted using simple slopes equations of the regression for conditional effects of perceived support at three levels, for men (**left**) and women (**right**).

The conditional interaction between neuroticism × perceived friend support and loneliness (indirect path a7i) was significant for men (adjusted *p* = 0.029) and not for women (adjusted *p* = 0.398). Conditional effects of neuroticism on loneliness were significant at each level of friend support for both men and women, with higher neuroticism and less perceived support associated with greater loneliness (see Supporting Information Table S3). At low and mean levels of perceived friend support, men scoring low on neuroticism reported greater loneliness than women. We observed the largest magnitude of effect in men when perceived support from friends was high, indicating that in men, the perception of greater friend support was less able to moderate the negative effects of neuroticism on loneliness than in women (Figure 2a).

A significant three-way interaction was also seen between neuroticism and well-being impact (direct path c7′). The conditional interaction between neuroticism × perceived friend support on well-being impact was significant for men (adjusted *p* = 0.015) but not significant for women (adjusted *p* = 0.062). For men, conditional effects of neuroticism on the impact of the pandemic on well-being were significant only at low values of perceived friend support, whilst for women, conditional effects were seen at all levels of friend support (See Supporting Information Table S4). Opposing crossover effects were observed for men and women. At low levels of perceived friend support, men showed a significant positive association of higher neuroticism on greater impact on well-being, whilst at high levels of perceived friend support, the relationship between neuroticism and well-being impact showed a non-significant negative association with almost no variation (Figure 2b). For women, at each level of friend support, higher levels of neuroticism were consistently associated with a greater impact on well-being, including a significant positive association between neuroticism and impact on well-being when perceived friend support was high.

## 4. Discussion

The goal of the current study was to examine the psychosocial mechanism of loneliness as a mediator between personality and health, subject to moderation by perceived social support and gender, within the context of the social and physical restrictions of the first pandemic lockdown in the UK. As hypothesised, evidence was found for loneliness as a mediator of personality on self-rated health, specifically, as a mediator between extraversion and general health and well-being impact, and between neuroticism and general health. Contrary to hypotheses, loneliness did not significantly mediate the relationship between neuroticism and the impact of the pandemic on well-being. Examination of perceived social support and gender as moderators of loneliness-mediated relationships between personality and health supported our hypothesis that perceived social support would act as a buffer of these mediation effects with differentiation by gender. Moderated mediation effects were observed only in the neuroticism–loneliness–well-being impact model, where perceived social support from friends moderated this relationship in men but not in women. Examination of specific moderation paths highlighted that with respect to loneliness, men who had a higher score on neuroticism were less able to benefit from the perception of greater support from friends compared to women; with respect to well-being, men who were higher on neuroticism were less sensitive than women to the negative impact of perceived low levels of friend support.

In comparison with women, men scored significantly lower on neuroticism and reported lower levels of perceived social support from all sources, although this difference was significant only for significant other and friend support. Both men and women ranked significant other as the greatest source of support, followed by friends and then family. This accords with findings reported for women’s friend support [38,39] and work in patients with a depressive disorder where women reported higher levels of significant other support than men [40]. Whilst there was no significant difference between men and women in self-rated general health or loneliness, women reported a significantly greater impact of the pandemic lockdown on their well-being, consistent with recent reports highlighting specific sociodemographic groups as being more vulnerable during the coronavirus pandemic, including women and young adults in the UK and internationally [14,17,18,21,22].

Unstratified by gender, higher scores on extraversion were indirect, via loneliness, associated with better self-rated general health and less of an impact of the pandemic on well-being, whereas higher scores on neuroticism were indirectly associated with poorer self-rated general health but not with impact on well-being. Consistent with work linking loneliness and the personality characteristics of extraversion and neuroticism [31], this supports the notion that neuroticism incurs an innate sensitivity to detect and respond to negative social stimuli that may increase feelings of loneliness and negative health outcomes [32]. Recent research during the pandemic lockdown has reported negative rather than expected positive relationships between the impact of the situation on daily life and loneliness [13], interpreted as a positive consequence of restrictions on increased support and cohesion during difficult times. The current findings view loneliness from the perspective of a mediator and include personality as a driving factor in the effects of loneliness on self-rated health. Loneliness was found only to mediate the relationship between neuroticism and self-rated general health, similar to other findings reported [32], but not between neuroticism and well-being impact specific to the pandemic. It is possible that loneliness has a less complex relationship as a mediator of extraversion on self-reported health and neuroticism on physical health than as a mediator of neuroticism on well-being, particularly under socially stressful circumstances. Findings of the moderated mediation analyses support this interpretation as the neuroticism–loneliness–well-being mediation model was significant only when perceived friend support and gender were included as moderators. That perceived support and gender acted as moderators of the indirect effect of neuroticism on well-being impact via loneliness furthers understanding of how social support perception is operationalised across genders and becomes differentially translated with respect to self-rated well-being effects. Findings indicate that for men higher on the personality dimension of neuroticism, the impact of the pandemic on their well-being was worse if they perceived themselves to have greater support, and these effects were mediated through feelings of loneliness. This gendered perspective adds to previous work that has controlled for sex in the analysis of relationships between neuroticism, loneliness and subjective health [32] rather than including it as a variable of interest and sheds light on important differences.

Whilst there is a large body of evidence in which perceived social support has been acknowledged as a key psychosocial factor influencing health outcomes, the mechanisms involved in this health-providing relationship are largely unclear [4], and social support is noted to have “one of the messiest literatures in psychology” [7] (p. 276). Findings in the current study are relevant within the framework of social safety theory [7] and align with other work examining processes involved in the balance and regulation of emotion in response to the pandemic [58]. Consistent with previous work that has highlighted the importance of considering the role of neuroticism in both mortality and mental and physical morbidity [59], an examination of specific component paths found the personality dimension of neuroticism to interact with the perception of friend support to influence loneliness and well-being. Further gendered effects were found in these analyses, which extend previous work on neuroticism and help to elucidate the differential mechanisms by which it might exert an influence. Compared to women, men scoring higher on neuroticism benefitted less from the perception of greater friend support, showing higher levels of loneliness, yet in relation to their well-being, they also showed comparatively lower sensitivity to a perceived lack of friend support. Consistent with previous work [41], women reported higher levels of all types of perceived social support than men, and this was particularly so for friend support. Yet, the potential beneficial effect of this on loneliness and well-being showed greater nuance than previously reported findings in which men tended to benefit more from some social relationships than women [42]. In the current study, it was the lack of sensitivity to low levels of friend support rather than the benefits of its presence that was associated with health impact. When taken together, our findings suggest that men who are higher on neuroticism are less affected by both the positive and negative effects of perceived friend support with respect to loneliness and well-being. There appears to be a gendered difference in those scoring higher on neuroticism, in which men function as social barometers to a lesser degree than women, and this is associated with the negative outcome of greater loneliness but also the positive outcome of better well-being. Whilst stress was not directly measured in this study, findings fit within the classic stress-buffering hypothesis of social support [60]. Having greater perceived friend support buffered the heightened sensitivity to negative social stimuli characteristic of neuroticism but to a lesser extent in men compared to women; perceived friend support was less able to buffer the socially sensitive characteristic of neuroticism against the experience of loneliness in men. The counterpart of this buffering insensitivity is consistent with what some authors [25] refer to as the social aggravation effect, in which social support fails or where support is itself a cause of stress. In the current findings, men who were higher in neuroticism were more able to resist the negative impacts of higher levels of perceived friend support as well as perceived low levels of friend support, whereas for women high in neuroticism, perceiving that they had more friend support was associated with a greater impact on their well-being. This suggests that women experienced social aggravation to a greater degree than men and that men were less sensitive to the negative effects of perceived low levels of friend support. These findings emphasise the need to include gender differences when untangling the complexity of social support perception on loneliness and well-being and to challenge assumptions about the benefits of support and whom the support may benefit, particularly with respect to the source of support.

### Limitations and Future Work

Whilst moderated mediation analyses enabled an in-depth exploration of gendered effects of perceived social support and loneliness in the COVID-19 context, it is acknowledged that the cross-sectional design of this study limits the strength of inferences that can be drawn. Longitudinal work is needed to examine such effects over time and across contexts in which support restrictions may provide insight into the effects of perceived support on health. More specifically, a comparison between these findings that focus on the start of the pandemic and subsequent effects at a later stage during the post-pandemic return to relative normalcy would be of value. Furthermore, a population containing broader ethnic and racial identity should be considered in future work. In the current study, our attention was on younger adults, given the importance of this age group with respect to loneliness and opportunities for intervention, but this also limits the findings. Further work is needed that examines moderation by gender and perceived support on personality–loneliness–health and well-being across different waves of the lifespan to examine age-specific relevance and whether these relationships are dynamic over time and amenable to change. This work was conducted during a viral pandemic which provided a petri-dish for examination of biopsychosocial effects on health and well-being. However, due to the nature of physical distancing and necessary social separation, the use of social support assessment that relates to the frequency and type of social interactions that are often presumed to be in-person was not feasible. A comparison of gendered effects of received as well as perceived support is needed to understand fully the relationships suggested here. The fact that participants received remuneration during a time of potential financial hardship may also have influenced the population sampled, although this is unlikely given the modest amount provided. Despite the proven predictive power of self-report single-item measures in longitudinal work in particular, this study was limited by the use of brief self-report health outcome measures. Future work that uses a greater range of more extensive health outcome measures, including biological markers of disease outcome, is warranted to assess the significance of these findings.

## 5. Conclusions

The findings of this study provide insight into the gendered effects of perceived support on health and well-being, highlighting the role of perceived friend support in relation to the experience of loneliness as a mediator of personality on well-being. Results suggest that situations such as that seen in the recent pandemic lockdown challenge understanding of the mechanisms through which social support becomes operationalised. The gender differences noted in regard to neuroticism suggest that men and women function as differential social barometers. Men higher on neuroticism experienced the disadvantage of being less able to benefit from lower loneliness when they perceived greater support from friends, yet the advantage of being less sensitive to the negative impact on their well-being when they perceived low levels of friend support. Findings provide valuable theoretical insight with the potential to inform interventions to reduce loneliness and improve health and well-being.

## Figures and Tables

**Table 1:** Sociodemographic Characteristics of the Sample (*n* = 530).

Variable	Mean (SD)	%	*n*
Age (years)	25.42 (4.13)	-	-
Gender identity ^b^			
Female		61.3	325
Male		38.7	205
Education ^a^			
Below degree level		35.1	186
Undergraduate degree		43.2	229
Postgraduate level		20.6	109
Prefer not to say		1.1	6
Ethnicity			
White (British, Irish, other)		82.8	439
White Mixed/multiple		3.5	27
Asian (Indian, Pakistani, Bangladeshi, Chinese)		8.8	47
Black/African/Caribbean		3.1	16
Other		0.2	1
Country of residence			
United Kingdom		66.8	354
England		27.5	146
Scotland		3.2	17
Wales		1.5	8
Northern Ireland		0.9	5
Living situation			
Live alone		6.8	36
Live with partner		41.7	221
Live with family		37.9	201
Live with friends/shared accommodation		9.6	51
Other		4.0	21
Relationship status			
Single		30.9	164
Casual relationship		7.0	37
Serious relationship		61.7	327
Prefer not to say		0.4	2
Currently isolating due to COVID-19 ^a^ Yes		30.2	160
Considered a key worker Yes		21.3	113

^a^ Indicates significant differences of characteristic on dependent variable of self-reported general health (education, *p* < 0.001; isolating, *p* = 0.004) and ^b^ impact on well-being (*p* < 0.001).

**Table 2:** Descriptive data for psychosocial and health outcome variables by gender.

Variable	Mean (SD)		*t* (528)	*p*
	Women (*n* = 325)	Men (*n* = 205)		
Personality				
Extraversion	29.15 (8.78)	28.57 (8.63)	0.756	0.450
Neuroticism	31.61 (7.97)	27.80 (8.05)	5.35	<0.001
Loneliness	46.21 (10.40)	46.29 (10.41)	−0.87	0.931
Perceived social support				
Total	5.36 (1.22)	5.15 (1.25)	1.83	0.068
Significant other	5.63 (1.61)	5.33(1.71)	2.02	0.044
Family	5.08 (1.54)	5.05(1.60)	0.18	0.854
Friends	5.36 (1.44)	5.08 (1.37)	2.22	0.027
Health				
General	3.49 (0.87)	3.57 (0.95)	−1.01	0.313
Impact on well-being	62.89 (21.31)	52.49 (24.72)	4.97	<0.001

**Table 3:** Intercorrelations of key study variables disaggregated by gender.

Variable	1	2	3	4	5	6	7	8	9
1. Extraversion	_	−0.30 ***	0.23 ***	0.15 **	0.12 *	0.30 ***	−0.43 ***	0.15 **	0.09
2. Neuroticism	−0.34 ***	_	−0.26 ***	−0.13 *	−0.31 ***	−0.18 ***	0.55 ***	−0.38 ***	0.40 ***
3. Support total	0.31 ***	−0.44 ***	_	0.81 ***	0.81 ***	0.77 ***	−0.64 ***	0.24 ***	−0.05
4. Support SO	0.21 **	−0.260 ***	0.82 ***	_	0.47 ***	0.43 ***	−0.48 ***	0.14 **	−0.00
5. Support Family	0.24 **	−0.380 ***	0.82 ***	0.47 ***	_	0.45 ***	−0.46 ***	0.24 ***	−0.11 *
6. Support Friends	0.32 ***	−0.440 ***	0.77 ***	0.45 ***	0.48 ***	_	−0.59 ***	0.20 ***	−0.01
7. Loneliness	−0.47 ***	0.650 ***	−0.71 ***	−0.51 ***	−0.55 ***	−0.66 ***	_	−0.32 ***	0.20 ***
8. Health General	0.18 **	−0.380 ***	0.24 **	0.13	0.28 ***	0.17 *	−0.28 ***	_	−0.26 ***
9. Well-being impact	−0.04	0.28 ***	−0.09	−0.05	−0.08	−0.10	0.26 ***	−0.22 **	_

Results for females (*n* = 325) are shown above the diagonal; results for males (*n* = 205) are shown below the diagonal. * *p* ≤ 0.05, ** *p* ≤ 0.01, *** *p* ≤ 0.001.

**Table 4:** Coefficients Across Models for Moderation Effects of Support × Gender on Specific Paths.

	Consequent Variable = Loneliness (*M*) (Specific Path a_7i_)							
Antecedent Variables (*X* × *W* × *Z*)	Unadjusted				Adjusted ^a^			
Neuroticism	*b* (*SE*)	*t*	95% CI	*p*	*b* (*SE*)	*t*	95% CI	*p*
**× Support Total ** **× Gender**	**0.122 (0.055)**	**2.228**	**[0.015, 0.230]**	**0.026**	0.106 (0.055)	1.914	[−0.003, 0.215]	0.056
× Support SO × Gender	0.081 (0.049)	1.666	[−0.015, 0.177]	0.096	0.067 (0.049)	1.357	[−0.030, 0.163]	0.175
× Support Family × Gender	0.035 (0.051)	0.681	[−0.065, 0.135]	0.496	0.017 (0.051)	0.334	[−0.084, 0.118]	0.739
**× Support Friend ** **× Gender**	**0.114 (0.051)**	**2.240**	**[0.014, 0.215]**	**0.026**	**0.116 (0.052)**	**2.232**	**[0.014, 0.218]**	**0.026**
**Consequent variable = Impact on Well-Being (*Y*) (specific path** **c_7′_)**								
× Support Total × Gender	−0.103 (0.233)	−0.443	−0.561 to 0.354	0.658	−0.216 (0.237)	−0.910	[−0.682 to 0.250]	0.363
× Support SO × Gender	0.255 (0.177)	1.442	−0.092 to 0.602	0.150	0.202 (0.178)	1.130	[−0.149 to 0.553]	0.259
× Support Family × Gender	−0.074 (0.173)	−0.429	−0.414 to 0.266	0.668	−0.131 (0.175)	−0.750	[−0.474 to 0.212]	0.454
**× Support Friend × Gender**	**−0.504 (0.203)**	**−2.482**	**−0.904 to −0.105**	**0.013**	**−0.628 (0.206)**	**−3.051**	**[−1.033 to −0.224]**	**0.002**

SO = Significant other. ^a^ Adjusted for covariates of age, education level and isolating status. Statistically significant interactions presented in bold.

## Data Availability

The data that support the findings of this study are openly available in Bournemouth University’s Online Research Data Repository (BORDaR) [61] at https://doi.org/10.18746/bmth.data.00000168, accessed on 5 June 2022.

## Supplementary Materials

The following supporting information can be downloaded at: https://www.mdpi.com/article/10.3390/ijerph19137986/s1, Table S1: Pairwise contrasts; Table S2: Conditional indirect effects; Table S3: Conditional effects on loneliness; Table S4: Conditional effects on well-being; Figure S1: Path coefficients for simple mediation; Figure S2: Full statistical model for moderated mediation analysis.

## References

[1] Ozbay F., Johnson D.C., Dimoulas E., Morgan C.A., Charney D., Southwick S. (2007). Social support and resilience to stress: From neurobiology to clinical practice. Psychiatry.

[2] House J.S., Landis K.R., Umberson D. (1988). Social relationships and health. Science.

[3] Begen F.M., Turner-Cobb J.M. (2015). Benefits of belonging: Experimental manipulation of social inclusion to enhance psychological and physiological health parameters. Psychol. Health.

[4] Uchino B.N., Bowen K., Carlisle M., Birmingham W. (2012). Psychological pathways linking social support to health outcomes: A visit with the “ghosts” of research past, present, and future. Soc. Sci. Med..

[5] Chen Y., Hicks A., While A.E. (2014). Loneliness and social support of older people in China: A systematic literature review. Health Soc. Care Community.

[6] Yildiz M.A., Karadas C. (2017). Multiple mediation of self-esteem and perceived social support in the relationship between loneliness and life satisfaction. J. Educ. Pract..

[7] Slavich G.M. (2020). Social safety theory: A biologically based evolutionary perspective on life stress, health, and behavior. Annu. Rev. Clin. Psychol..

[8] World Health Organisation (2020). Virtual Press Conference on COVID-19. https://www.who.int/docs/default-source/coronaviruse/transcripts/who-audio-emergencies-coronavirus-press-conference-full-and-final-11mar2020.pdf?sfvrsn=cb432bb3_2.

[9] Linton N.M., Kobayashi T., Yang Y., Hayashi K., Akhmetzhanov A.R., Jung S., Yuan B., Kinoshita R., Nishiura H. (2020). Incubation period and other epidemiological characteristics of 2019 novel coronavirus infections with right truncation: A statistical analysis of publicly available case data. J. Clin. Med..

[10] Shereen M.A., Khan S., Kazmi A., Bashir N., Siddique R. (2020). COVID-19 infection: Origin, transmission, and characteristics of human coronaviruses. J. Adv. Res..

[11] Ahmed F., Zviedrite N., Uzicanin A. (2018). Effectiveness of workplace social distancing measures in reducing influenza transmission: A systematic review. BMC Public Health.

[12] Brooks S.K., Webster R.K., Smith L.E., Woodland L., Wessely S., Greenberg N., Rubin G.J. (2020). The psychological impact of quarantine and how to reduce it: Rapid review of the evidence. Lancet.

[13] Tull M.T., Edmonds K.A., Scamaldo K.M., Richmond J.R., Rose J.P., Gratz K.L. (2020). Psychological outcomes associated with stay-at-home orders and the perceived impact of COVID-19 on daily life. Psychiatry Res..

[14] Bu F., Steptoe A., Fancourt D. (2020). Loneliness during a strict lockdown: Trajectories and predictors during the COVID-19 pandemic in 38,217 United Kingdom adults. Soc. Sci. Med..

[15] Cacioppo J.T., Cacioppo S. (2014). Social relationships and health: The toxic effects of perceived social isolation. Soc. Personal. Psychol. Compass.

[16] Leigh-Hunt N., Bagguley D., Bash K., Turner V., Turnbull S., Valtorta N., Caan W. (2017). An overview of systematic reviews on the public health consequences of social isolation and loneliness. Public Health.

[17] Kowal M., Coll-Martin T., Ikizer G., Rasmussen J., Eichel K., Studzinska A., Koszalkowska K., Karwowski M., Najmussaqib A., Pankowski D. (2020). Who is the most stressed during the COVID-19 pandemic? Data from 26 countries and areas. Appl. Psychol. Health Well-Being.

[18] Pieh C., Budimir S., Delgadillo J., Barkham M., Fontaine J.R.J., Probst T. (2021). Mental Health During COVID-19 Lockdown in the United Kingdom. Psychosom. Med..

[19] Killgore W.D.S., Cloonan S.A., Taylor E.C., Dailey N.S. (2020). Loneliness: A signature mental health concern in the era of COVID-19. Psychiatry Res..

[20] Liu C.H., Zhang E., Wong G.T.F., Hyun S., Hahm H.C. (2020). Factors associated with depression, anxiety, and PTSD symptomatology during the COVID-19 pandemic: Clinical implications for U.S. young adult mental health. Psychiatry Res..

[21] Bu F., Steptoe A., Fancourt D. (2020). Who is lonely in lockdown? Cross-cohort analyses of predictors of loneliness before and during the COVID-19 pandemic. Public Health.

[22] O’Connor R.C., Wetherall K., Cleare S., McClelland H., Melson A.J., Niedzwiedz C.L., O’Carroll R.E., O’Connor D.B., Platt S., Scowcroft E. (2021). Mental health and well-being during the COVID-19 pandemic: Longitudinal analyses of adults in the UK COVID-19 Mental Health & Wellbeing study. Br. J. Psychiatry.

[23] Li F., Luo S., Mu W., Li Y., Ye L., Zheng X., Xu B., Ding Y., Ling P., Zhou M. (2021). Effects of sources of social support and resilience on the mental health of different age groups during the COVID-19 pandemic. BMC Psychiatry.

[24] Ye Z., Yang X., Zeng C., Wang Y., Shen Z., Li X., Lin D. (2020). Resilience, Social Support, and Coping as Mediators between COVID-19-related Stressful Experiences and Acute Stress Disorder among College Students in China. Appl. Psychol. Health Well-Being.

[25] Birmingham W.C., Holt-Lunstad J. (2018). Social aggravation: Understanding the complex role of social relationships on stress and health-relevant physiology. Int. J. Psychophysiol..

[26] Harada K., Sugisawa H., Sugihara Y., Yanagisawa Y., Shimmei M. (2018). Social support, negative interactions and mental health: Evidence of cross-domain buffering effects among older adults in Japan. Res. Aging.

[27] Lincoln K.D. (2000). Social support, negative social interactions and psychological wellbeing. Soc. Serv. Rev..

[28] Tian Q. (2016). Intergeneration social support affects the subjective well-being of the elderly: Mediator roles of self-esteem and loneliness. J. Health Psychol..

[29] Asendorpf J.B., Wilpers S. (1998). Personality effects on social relationships. J. Pers. Soc. Psychol..

[30] McCrae R.R., John O.P. (1992). An Introduction to the Five-Factor Model and Its Applications. J. Pers..

[31] Abdellaoui A., Chen H.-Y., Willemsen G., Ehli E.A., Davies G.E., Verweij K.J.H., Nivard M.G., De Geus E.J.C., Boomsma R.I., Cacioppo J.T. (2019). Associations between loneliness and personality are mostly driven by a genetic association with neuroticism. J. Pers..

[32] Mund M., Neyer F.J. (2016). The Winding Paths of the Lonesome Cowboy: Evidence for Mutual Influences Between Personality, Subjective Health, and Loneliness. J. Pers..

[33] Lakey B., Orehek E. (2011). Relational regulation theory: A new approach to explain the link between perceived social support and mental health. Psychol. Rev..

[34] Norris F.H., Kaniasty K. (1996). Received and perceived social support in times of stress: A test of the social support deterioration deterrence model. J. Pers. Soc. Psychol..

[35] Haden S.C., Scarpa A., Jones R.T., Ollendick T.H. (2007). Posttraumatic stress disorder symptoms and injury: The moderating role of perceived social support and coping for young adults. Pers. Individ. Differ..

[36] Cohen S., Mermelstein R., Kamarck T., Hoberman H.M. (1985). Measuring the functional components of social support. Social Support: Theory, Research and Applications.

[37] Sarason I.G., Sarason B.R., Shearin E.N., Pierce G.R. (1987). A Brief Measure of Social Support: Practical and Theoretical Implications. J. Soc. Pers. Relatsh..

[38] Harrison J., Maguire P., Pitceathly C. (1995). Confiding in crisis: Gender differences in pattern of confiding among cancer patients. Soc. Sci. Med..

[39] Lynch S.A. (1998). Who supports whom? How age and gender affect the perceived quality of support from family and friends. Gerontologist.

[40] Soman S., Bhat S.M., Latha K.S., Praharaj S.K. (2016). Gender differences in perceived social support and stressful life events in depressed patients. East Asian Arch. Psychiatry.

[41] Tifferet S. (2020). Gender differences in social support on social network sites: A meta-analysis. Cyberpsychol. Behav. Soc. Netw..

[42] Murphy M., Grundy E., Kalogirou S. (2007). The increase in marital status differences in mortality up to the oldest age in seven European countries, 1990–1999. Popul. Stud..

[43] Phillips A.C., Gallagher S., Carroll D. (2009). Social support, social intimacy, and cardiovascular reactions to acute psychological stress. Ann. Behav. Med..

[44] Uhing A., Williams J.S., Garacci E., Egede L.E. (2021). Gender differences in the relationship between social support and strain and mortality among a national sample of adults. J. Behav. Med..

[45] Cascalheira C., McCormack M., Portch E., Wignall L. (2021). Changes in sexual fantasy and solitary sexual practice as a result of social lockdown among young adults in the UK. Sex. Med..

[46] Wignall L., Portch E., McCormack M., Owens R., Cascalheira C., Attard-Johnson J., Cole T. (2021). Changes in sexual desire and behaviors among UK young adults during social lockdown due to COVID-19. J. Sex Res..

[47] International Personality Item Pool (IPIP) (2019). A Scientific Collaboratory for the Development of Advanced Measures of Personality Traits and Other Individual Differences. http://ipip.ori.org/.

[48] Goldberg L.R., Johnson J.A., Eber H.W., Hogan R., Ashton M.C., Cloninger C.R., Gough H.C. (2006). The International Personality Item Pool and the future of public-domain personality measures. J. Res. Pers..

[49] Russell D.W. (1996). UCLA Loneliness scale (version 3): Reliability, validity, and factor structure. J. Pers. Assess..

[50] Zimet G.D., Dahlem N.W., Zimet S.G., Gordon K.F. (1988). The multidimensional scale of perceived social support. J. Pers. Assess..

[51] Clara I.P., Cox B.J., Enns M.W., Murray L.T., Torgrudc L.J. (2003). Confirmatory factor analysis of the multidimensional scale of perceived social support in clinically distressed and student samples. J. Pers. Assess..

[52] Osman A., Lamis D.A., Freedenthal S., Gutierrez P.M., McNaughton-Cassill M. (2014). The multidimensional scale of perceived social support: Analyses of internal reliability, measurement invariance, and correlates across gender. J. Pers. Assess..

[53] Ware J.E., Snow K.K., Kosinski M., Gandek B. (1993). The SF-36 Health Survey: Manual and Interpretation Guide.

[54] Cohen S., Janicki-Deverts D., Doyle W.J. (2015). Self-rated health in healthy adults and susceptibility to the common cold. Psychosom. Med..

[55] DeSalvo K.B., Bloser N., Reynolds K., He J., Muntner P. (2006). Mortality prediction with a single general self-rated health question. A meta-analysis. J. Gen. Intern. Med..

[56] Uchino B.N., Landvatter J., Cronan S., Scott E., Papadakis M., Smith T.W., Bosch J.A., Joel S. (2019). Self-rated health and inflammation: A test of depression and sleep quality as mediators. Psychosom. Med..

[57] Hayes A.F. (2018). Introduction to Mediation, Moderation, and Conditional Process Analysis: A Regression-Based Approach.

[58] Mariani R., Renzi A., Di Monte C., Petrovska E., Di Trani M. (2021). The impact of the COVID-19 pandemic on primary emotional systems and emotional regulation. Int. J. Environ. Res. Public Health.

[59] Lahey B.B. (2009). Public health significance of neuroticism. Am. Psychol..

[60] Cohen S., Wills T.A. (1985). Stress, social support, and the buffering hypothesis. Psychol. Bull..

[61] Wignall L., Portch E., Turner-Cobb J., Attard-Johnson J., Cole T., Cascalheira C., Owens R. (2021). Sexual Behaviours, Desires and Wellbeing of UK Young Adults during Social Lockdown Due to COVID-19.

