# Supplementary material for: Men and Women as Differential Social Barometers: Gender Effects of Perceived Friend Support on the Neuroticism-Loneliness-Well-Being Relationship in a Younger Adult Population
Source: Int J Environ Res Public Health. 2022 Jun 29;19(13):7986. doi: 10.3390/ijerph19137986 (PMC9265668; doi:10.3390/ijerph19137986)
Supplement: Supplementary file 1 [file ijerph-19-07986-s001.zip › ijerph-1782767-supplementary.pdf]

Supplementary Materials

**Table S1**	Pairwise contrasts between conditional indirect effects for Neuroticism-Loneliness-Impact of Well-being mediation model with Perceived friend support and Gender as moderators	Page 2
**Table S2**	Conditional indirect effects of neuroticism on well-being impact via loneliness at levels of perceived friend support and gender	Page 3
**Table S3**	Conditional Effects (Mean, ± 1SD) on loneliness of Neuroticism at Values of the Moderators	Page 3
**Table S4**	Conditional Effects (Mean, ± 1SD) on well-being impact of Neuroticism at Values of the Moderators	Page 4
**Figure S1**	Path Coefficients for Simple Mediation Analysis with Loneliness as a Mediator of Personality on Self-Reported Health	Page 5
**Figure S2**	Full statistical model for mediation of neuroticism on well-being impact via loneliness with perceived friend support and gender as moderators	Page 6−7

Table S1

Pairwise contrasts between conditional indirect effects for Neuroticism-Loneliness-Impact of Well-being mediation model with Perceived friend support and Gender as moderators.

Effect 1	Effect 2	Contrast (se)	95% Bootstrap CI
0.121	−0.002	0.122	[−0.273, 0.569]
0.049	−0.002	0.051	[−0.091, 0.215]
0.351	−0.002	0.353	[−0.007, 0.773]
0.093	−0.002	0.095	[−0.171, 0.408]
0.664	−0.002	0.666	[0.084, 1.249]
0.049	0.121	−0.072	[−0.481, 0.302]
0.351	0.121	0.230	[−0.026, 0.481]
0.093	0.121	−0.027	[−0.458, 0.375]
0.664	0.121	0.544	[−0.100, 1.146]
0.351	0.049	0.302	[−0.042, 0.685]
0.093	0.049	0.044	[−0.080, 0.200]
0.664	0.049	0.615	[0.051, 1.174]
0.093	0.351	−0.257	[−0.660, 0.114]
0.664	0.351	0.313	[−0.077, 0.676]
0.664	0.093	0.571	[−0.012, 1.150]

Note. Contrast = Effect 1 minus effect 2

CI = confidence interval.

Values show adjusted results with age, education level and isolation status included as covariates.

Table S2

Conditional indirect effects of neuroticism on well-being impact via loneliness at levels of perceived friend support and gender

Perceived friend support	Gender	*b* coefficient	Boot se	95% Bootstrap CI
−1.410	Women	−0.002	0.129	[−0.286, 0.231]
−1.410	Men	0.121	0.167	[−0.195, 0.450]
0.00	Women	0.049	0.109	[−0.175, 0.253]
0.00	Men	0.351	0.150	[0.063, 0.651]
1.410	Women	0.093	0.132	[−0.174, 0.356]
1.410	Men	0.664	0.271	[0.136, 1.194]

Note. Low, mean and high levels of perceived support are shown as 1SD below the mean, mean and +1SD above the mean.

CI = confidence interval.

Values show adjusted results with age, education level and isolation status included as covariates.

Table S3

Conditional Effects (Mean, ± 1SD) on loneliness of Neuroticism at Values of the Moderators

Moderator values		b	se	t	*p*	95% *CI*	
Support friends	Gender					LL	UL
−1.410	Women	0.649	0.069	9.431	<0.001	0.514	0.785
−1.410	Men	0.470	0.083	5.653	<0.001	0.307	0.634
0.00	Women	0.611	0.049	12.346	<0.001	0.514	0.708
0.00	Men	0.595	0.068	8.758	<0.001	0.462	0.729
1.410	Women	0.572	0.066	8.667	<0.001	0.442	0.702
1.410	Men	0.720	0.094	7.667	<0.001	0.536	0.905

Note: Gender values assigned as women = 0; men = 1.

Values show adjusted results when age, education level and isolation status are included as covariates.

Table S4

Conditional Effects (Mean, ± 1SD) on well-being impact of Neuroticism at Values of the Moderators

Moderator values		b	se	t	*p*	95% *CI*	
Support friends	Gender					LL	UL
−1.410	Women	0.742	0.247	3.007	=0.003	0.257	1.226
−1.410	Men	1.059	0.297	3.564	<0.001	0.475	1.643
0.00	Women	1.045	0.183	5.719	<0.001	0.686	1.403
0.00	Men	0.476	0.250	1.907	=0.057	−0.014	0.967
1.410	Women	1.348	0.242	5.568	<0.001	0.782	1.823
1.410	Men	−0.106	0.388	−0.274	=0.784	−0.870	0.657

Note: Gender values assigned as women = 0; men = 1.

Values show adjusted results when age, education level and isolation status are included as covariates.

Figure S1. Path Coefficients for Simple Mediation Analysis with Loneliness as a Mediator of Personality on Self-Reported Health

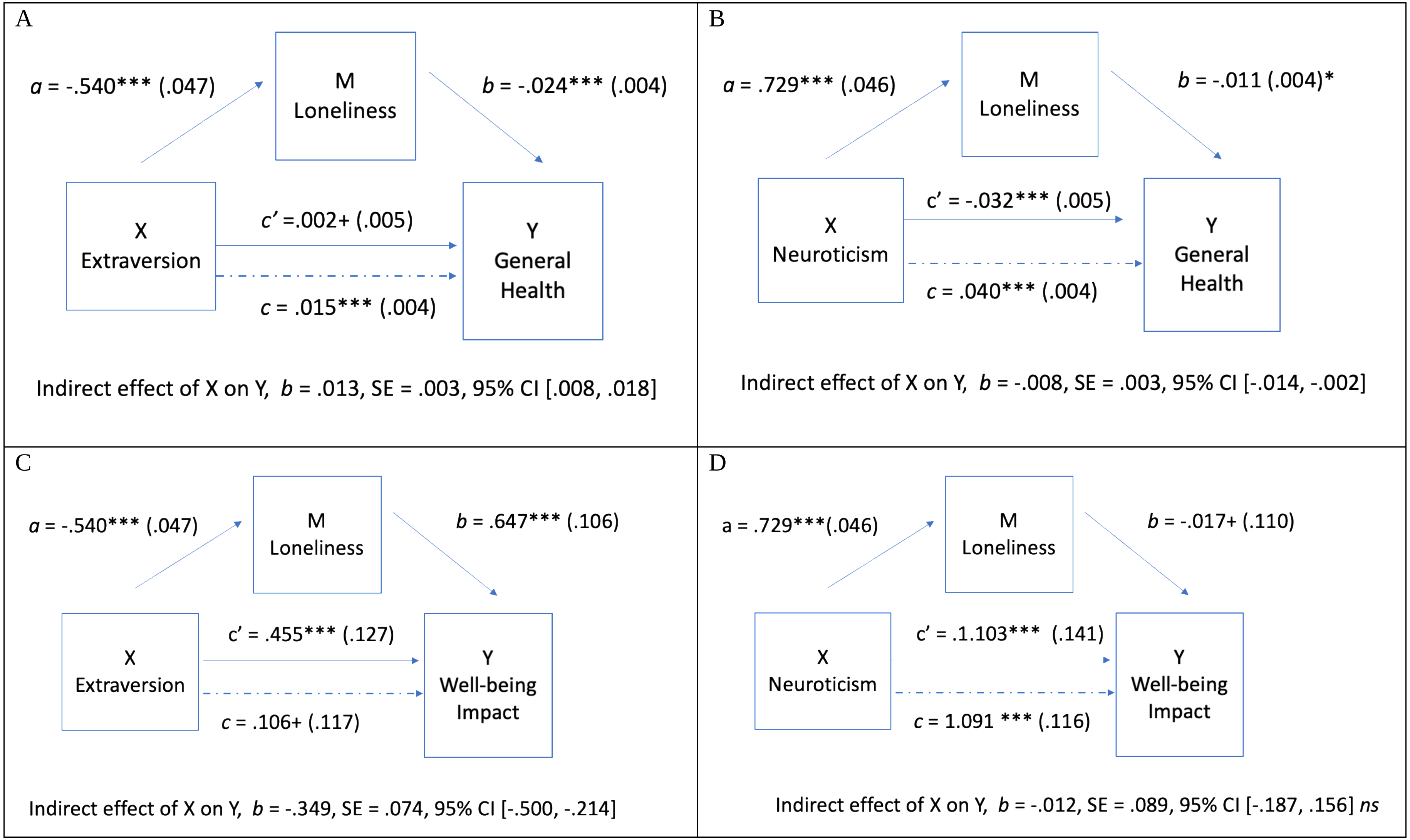

Note: Adjusted models shown with covariates of age, education level and isolation status

Figure S2

Full statistical model for mediation of neuroticism on well-being impact via loneliness with perceived friend support and gender as moderators

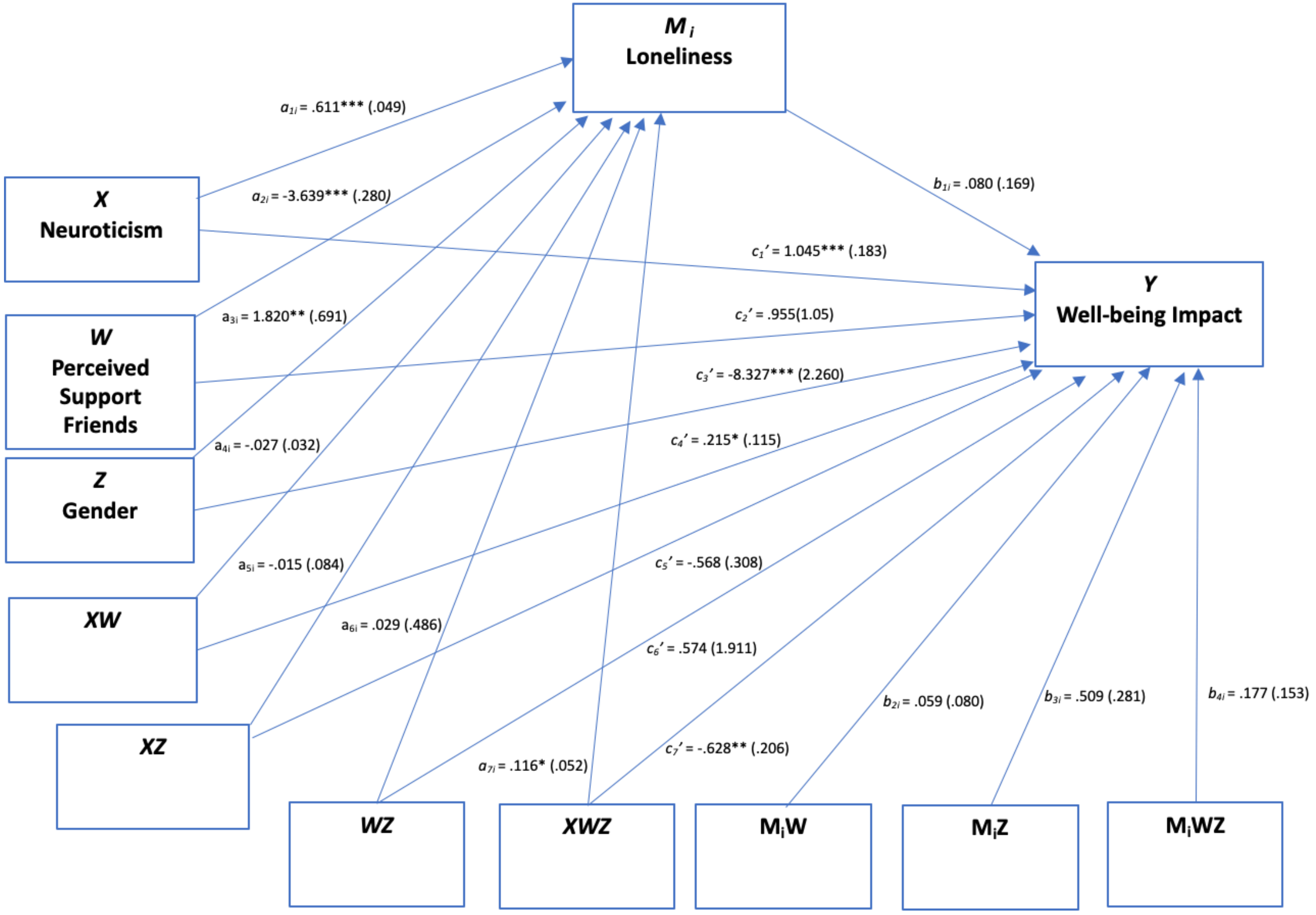

Note. Values show adjusted b coefficient (se) when age, education level and isolation status are included as covariates.

Covariate main effects with loneliness: age, education level, isolation, all ns; well-being impact: age, ns; education level, p =0.01; isolation status, $p = 0.046$

* $p \leq 0.05$, ** $p < 0.01$, *** $p < 0.001$
